# HIV-Associated Oral Mucosal Melanin Hyperpigmentation: A Clinical Study in a South African Population Sample

**DOI:** 10.1155/2016/8389214

**Published:** 2016-02-24

**Authors:** R. Chandran, L. Feller, J. Lemmer, R. A. G. Khammissa

**Affiliations:** Department of Periodontology and Oral Medicine, Sefako Makgatho Health Sciences University, Pretoria 0204, South Africa

## Abstract

*Objective*. The aim of the study was to determine the prevalence of HIV-associated oral mucosal melanin hyperpigmentation (HIV-OMH) in a specific population of HIV-seropositive South Africans and to analyse the associations between HIV-OMH clinical features and the demographic and immunological characteristics of the study cohort.* Material and Methods*. This cross-sectional study included 200 HIV-seropositive Black subjects. The collected data comprised age, gender, CD4+ T cell count, viral load, systemic disease, medications, oral site affected by HIV-OMH, extent (localized or generalized), intensity of the pigmentation (dark or light), and smoking and snuff use.* Results*. Overall, 18.5% of the study cohort had HIV-OMH. Twenty-two and a half percent had OMH that could not with confidence be attributed to HIV infection, and 59% did not have any OMH. There was a significant but weak association between smoking and the presence of HIV-OMH.* Conclusions*. The prevalence of HIV-OMH in the study population was 18.5%, the gingiva being the most commonly affected site. It appears that the CD4+ T cell count does not play any role in the biopathology of HIV-OMH.

## 1. Introduction

According to the EC-Clearinghouse Classification of Oral Lesions Associated with Adult HIV Infection of September 1992 [1], HIV-associated oral melanotic hyperpigmentation is categorised under “lesions less commonly associated with HIV infection” (Group 2). What is referred to in these documents as “HIV-associated oral melanotic hyperpigmentation,” we will term in this paper “HIV-associated oral mucosal melanin hyperpigmentation” (HIV-OMH) which we believe more accurately describes this condition.

**Figure 1:**
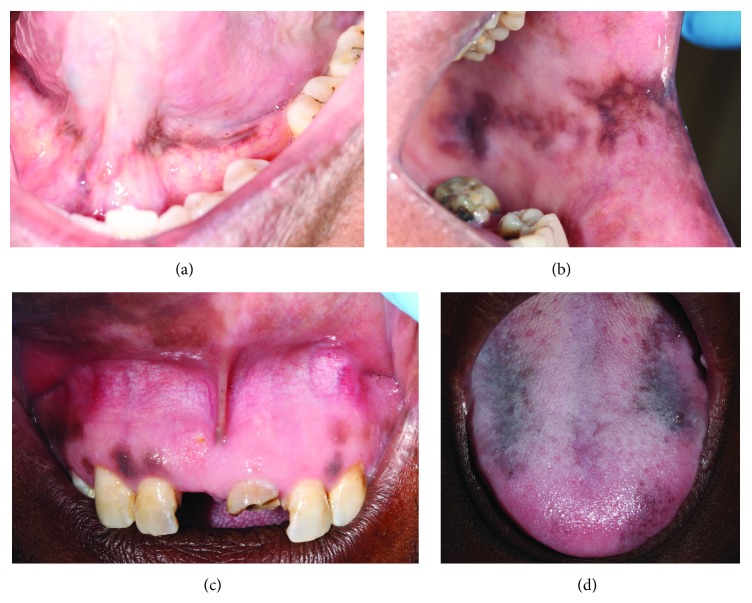
Clinical illustration of HIV-OMH. (a) Melanin hyperpigmentation of the floor of the mouth of a female 42-year-old nonsmoker who was HIV-seropositive with a CD4+ T cell count of 25 cells/mm^3^. She had been diagnosed with HIV disease 3 years previously. Highly active antiretroviral therapy (HAART) was started immediately after the diagnosis, and the hyperpigmentation appeared some time later. (b) Irregular, nonhomogeneous pigmented patch on the buccal mucosa of a 69-year-old HIV-seropositive male with a CD4+ T cell count of 88 cells/mm^3^. He had been diagnosed with HIV infection seven years previously. HAART was started after 2 years and the hyperpigmentation appeared 4 years later. The patient is a nonsmoker. (c) Multiple, pigmented maculae on the maxillary gingiva and labial mucosa of a 40-year-old HIV-seropositive male with a CD4+ T cell count of 141 cells/mm^3^. He had been diagnosed with HIV disease 10 years previously and HAART was started immediately. The hyperpigmentation appeared two years later. (d) Multiple pigmented maculae of the dorsum of the tongue in a 32-year-old HIV-seropositive male on HAART with a CD4+ T cell count of 422 cells/mm^3^. He had been found to be HIV-seropositive eight years previously and HAART was started a year later. The patient recalls that the hyperpigmentation developed after the start of HAART medication but he is unsure exactly when it appeared. None of the patients whose oral pigmentation is illustrated had any other oral soft tissue abnormalities or any known systemic disease.

HIV-OMH can affect any part of the oral mucosa and usually appears as asymptomatic, single or multiple, well or ill-defined, and light to dark brown maculae of variable size and shape (Figure 1) [2]. Its etiopathogenesis is obscure, but it has been suggested that agents that may play a role in the development of HIV-OMH include HIV-induced cytokine dysregulation, some of the drugs commonly used in the treatment of HIV disease, and adrenocortical dysfunction which not infrequently affects HIV-seropositive subjects with low CD4+ T cell counts [3].

The prevalence of HIV-OMH varies in different parts of the world and between different ethnic/racial groups [4], most probably owing to genetic and environmental factors, to specific biochemical, pathological, immune, or other characteristics of HIV disease, and also to the drug regimen used to treat the HIV disease and its complications [2, 5].

It appears that HIV-OMH does not have any effect on oral health or on quality of life, and it is unresearched and unknown whether or not HIV-OMH has any pathological significance. Further research is therefore required into the intra- and extracellular biological pathways involved in the upregulation of melanogenesis in some HIV-seropositive subjects and into possible microenvironmental stimuli that may drive the process [2].

The aim of this study was to determine the prevalence of HIV-OMH in a geographic-specific population of HIV-seropositive South Africans attending an HIV outpatient clinic. While this population does not represent the ethnic diversity of the entire South African population, our study provides an insight into the distribution and correlates of HIV-OMH with demographic, clinical, and immunological characteristics of the study cohort.

## 2. Materials and Methods

The protocol of this cross-sectional study was approved by the Research Ethics Committee of the University of Limpopo, Medunsa Campus [number MREC/D/78/2104]. Participants were given both verbal and written information in their mother tongues about the nature of the study, and written informed consent was obtained. All personal information about the patients was kept confidential.

The study cohort comprised 200 HIV-seropositive Black patients attending the outpatient clinic of the Infectious Diseases Unit at Tshepang Clinic, Dr George Mukhari Academic Hospital or at the Medunsa Oral Health Centre, Pretoria, South Africa. Most of the patients attending these health-care facilities are patients from the surrounding semiurban communities, and by chance all the subjects in the study population happened to be Black. The HIV status of all the subjects had previously been established by two enzyme-linked immunosorbent assays (ELISA-HIV). The study was conducted over a period of 6 months from February to August 2014.

One of the authors (RC), examined and interviewed all the subjects, recorded the relevant clinical data, and consulted the medical records for supplementary medical history. If present, HIV-OMH and any other possibly HIV-related oral lesions were classified according to the criteria of EC-Clearinghouse [1].

The collected data included age, gender, CD4+ T cell count, presence or history of any systemic disease, medication, presence of HIV-OMH and if present its extent (localized or generalized), and the intensity (dark or light). Tobacco smoking and snuff use were also recorded. As information about the period between the diagnosis of HIV disease and the initiation of HAART to the appearance of HIV-OMH was not always available or reliable, these factors were not included in the study.

The extent of HIV-OMH was categorised as localised or generalised according to the number of oral mucosal regions affected by the hyperpigmentation and according to the extent of the pigmentation within a particular region. The intensity of the pigmentation was subjectively dichotomised as light or dark. For any other oral lesion or condition requiring treatment that was observed, the patient was referred to the appropriate hospital department.

Statistical analysis between-group tests were conducted as follows: *χ* ^2^ test was used to assess the relationship between categorical variables. Fisher's exact test was used for 2 × 2 tables or where the requirements for *χ* ^2^ test could not be met. The strength of the associations was measured by Cramer's *V* and the phi coefficient, respectively.

The relationship between continuous and categorical variables was assessed by the *t*-test. Where the data did not meet the assumptions of the *t*-test, a nonparametric alternative, the Wilcoxon rank sum test was used. The strength of the associations was measured by Cohen's *d* and the *r*-value, respectively.

The 5% significance level was used.

## 3. Results

Two hundred HIV-seropositive subjects, 134 (67%) females and 66 (33%) males, were included in this study (F : M = 2.03). Their mean age was 41.6 years; 14.5% were smokers and 4% used snuff. Overall, the median CD4+ T cell count was 159 cells/mm^3^ (interquartile range of 57 to 304) and the median viral load was 40 (interquartile range of 40 to 9668). All the patients were on HAART, and there were 15 HAART combinations made up of 11 drugs.

**Figure 2:**
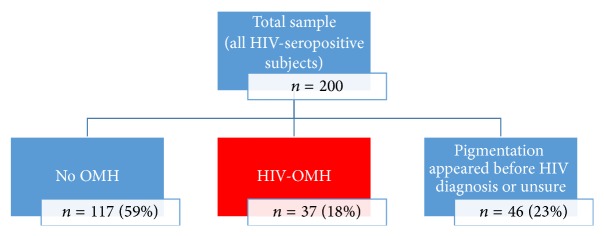
Grouping of the study subjects.

Of the study population of 200 HIV-seropositive subjects, 117 (59%) did not have any OMH (No OMH group), and 37 (18.5%) self-reportedly developed their oral hyperpigmentation after having been diagnosed as HIV-seropositive and thus were included in the HIV-OMH group. The remaining 46 study subjects (23%) either had self-reportedly developed OMH before HIV diagnosis (*n* = 26) or were unsure when the OMH had developed in relation to the diagnosis of HIV infection (*n* = 20) (Figure 2) and thus were not included in further analysis.

The mean age of the No OMH group (*n* = 117) was 40.9 years (sd = 9.9; range 34–47; median = 40 years) whereas the mean age of the HIV-OMH group (*n* = 37) was 42.5 years (sd = 10.7 y; range 36–48 y; median = 42 years) (Table 1). There was no significant difference in the mean age or in the age categories between the No OMH group and the HIV-OMH group. The No OMH group comprised 77.8% females (F : M = 3.5 : 1) whereas the HIV-OMH group comprised 56.7% females (F : M = 1.31 : 1) (Table 1). There was a significant but weak association between the two groups with regard to gender (*p* = 0.019; phi coefficient = 0.20).

There was a significant but weak association between HIV-OMH and smoking (*p* = 0.019; phi coefficient = 0.28). Two point six percent of the No OMH group were smokers compared to 18.9% of the HIV-OMH group (Table 1).

There was no significant association between snuff use, systemic disease, duration of HIV infection, HAART regimen, duration of use of HAART, CD4+ T cell count, and viral load on the one hand, and the presence or absence of HIV-OMH on the other hand.

The frequencies, with which the oral sites were affected, and the chief clinical characteristics of HIV-OMH are shown in Table 2. Within the HIV-OMH group, there was no significant association either between the CD4+ T cell counts (0–199, 200–499, >499) or between smoking and the extent, intensity, or site affected by OMH. However, the size of the HIV-OMH group was very small (*n* = 37) and therefore these results need to be interpreted with caution.

## 4. Discussion

The normal colour of the oral mucosa is determined by several factors including the thickness and transparency of the epithelium, whether the epithelium is para- or orthokeratinised, the vascularity of the lamina propria, the blood haemoglobin level, and the amount and colour of the melanin in the oral epithelium [6–8].

OMH may be racial/physiological or can occur in association with endocrine disorders (Addison disease, acromegaly), immunoinflammatory processes (oral lichen planus), and infections (HIV infection) or may represent a primary neoplasm (melanoma) [8].

The contribution of melanin to the colour of the oral mucosa is determined by the inherent baseline level of activity of enzymes and proteins which drive the process of melanin biosynthesis, and the magnitude of the response of melanocytes to extrinsic melanogenic stimuli, which is dependent on the functional activity of ligand-receptor interactions and intracellular signalling pathways [9, 10]. Further factors contributing to the colour of the oral mucosa include the number and the metabolic activity of epithelial melanocytes, the melanogenic activity of the melanocytes and the type of melanin produced, the size and number of the melanosomes, the extent of arborisation of the melanocytic dendritic processes, and the efficacy with which the melanosomes are transferred from the melanocytic dendritic processes to the surrounding keratinocytes within the keratinocyte-melanocyte unit.

Thus, the process of OMH is complex and the specific mechanisms that drive it in the context of HIV infection are unknown. It appears that HIV-OMH is the result of increased melanogenesis by melanocytes in the basal cell layer of the epithelium without an increase in the number of melanocytes, but the increased amount of melanin can be observed in the epithelium, in the lamina propria, or in both [11].

It is possible that the upregulation of IL-1, IL-6, and TNF-*α* associated with HIV infection triggers keratinocytes and melanocytes to produce alpha melanocyte stimulating hormone (*α*MSH) which has the capacity to stimulate melanogenesis, resulting in increased production of melanin, manifesting clinically as HIV-OMH (Figure 1). In HIV-seropositive subjects, OMH may also be induced by drugs often taken for the treatment of HIV infection and of HIV-associated systemic conditions [2]. In fact, there is some evidence that the prevalence of OMH is higher in HIV-seropositive subjects on HAART than in HIV-seropositive HAART-naïve subjects [12, 13].

The prevalence of HIV-OMH in our study population sample was 18.5%, significantly higher than in other countries in sub-Saharan Africa (Tanzania 4.7%, Kenya 6%) [14, 15] and in Europe (Italy 6.4%, Greece 2%) but lower than in Venezuela (38%) [16] and in India (26% to 35%) [17, 18]. To the best of our knowledge there is only one other report from South Africa documenting HIV-OMH, and in this report the prevalence of HIV-OMH was less than 1% [19]. However, this study was done in a completely different South African population comprising largely persons of Asian and mixed racial/ethnic descent, and the authors acknowledged that sometimes they could not determine with confidence whether the onset of the OMH did or did not follow the onset of HIV disease and associated medications.

It has also been reported that at the time of the studies, the frequency of HIV-OMH was inversely proportional to the CD4+ T cell count [3] and that HIV-OMH affects the buccal mucosa more commonly than any other oral sites [3, 20]. Our study does not confirm these observations, as we found no significant association between HIV-OMH and either the oral site affected or the CD4+ T cell count. The only significant association that we found was that HIV-seropositive subjects who smoked more frequently had OMH than those who did not.

## Figures and Tables

**Table 1:** Demographic and immunological characteristics of the study population; all subjects were Black.

		No OMH (*n* = 117)		HIV-OMH (*n* = 37)	
Age group	≤30 years	16	13.68%	3	8.11%
	31–40 years	49	41.88%	12	32.43%
	41–50 years	33	28.21%	15	40.54%
	>50 years	19	16.24%	7	18.92%

Gender	Female	91	77.78%	21	56.76%
	Male	26	22.22%	16	43.24%

Smoking status	No	114	97.44%	30	81.08%
	Yes	3	2.56%	7	18.92%

Snuff use	No	114	97.44%	34	91.89%
	Yes	3	2.56%	3	8.11%

CD4+ T cell count (cells/mm^3^)	0–199	71	60.68%	21	56.76%
	200–499	32	27.35%	12	32.43%
	>499	13	11.11%	4	10.81%
	Unknown	1	0.85%	0	0.00%

Viral load	≤1000	75	64.10%	24	64.86%
	>1000	42	35.90%	13	35.14%

**Table 2:** HIV-OMH: sites affected and clinical characteristics.

	Clinical parameters	HIV-OMH (*n* = 37)	
Intraoral site	Gingiva	17	45.95%
	Buccal mucosa	11	29.73%
	Palate-hard	6	16.22%
	Palate-soft	2	5.41%
	Tongue	6	16.22%
	Labial mucosa	7	18.92%
	Floor of the mouth	1	2.70%
	Alveolar ridge	1	2.70%

Extent	Localised/single	3	8.11%
	Generalised/multiple	34	91.89%

Intensity	Light	20	54.05%
	Dense	17	45.95%

## Comments

Investigating epidemiological and clinical features of HIV-OMH in general is difficult and in South African populations particularly so, because Black South Africans are known to have a high prevalence of physiological/racial hyperpigmentation, and, in HIV-seropositive subjects, differentiation between this and HIV-OMH relies on self-reported histories that are not always reliable. Furthermore as OMH is asymptomatic and sometimes affected oral sites are not readily visible to the subjects themselves, a substantial number of subjects cannot even state with confidence whether or not they have OMH, far less whether or not it was present before or after their diagnosis of HIV infection.

It is not uncommon in rural and semirural areas in South Africa for HIV infection to be diagnosed and treatment started late in the course of the disease. As a consequence, many subjects report with confidence that their OMH was present before their HIV infection was diagnosed, when in fact they may well already have been HIV-seropositive when they first noticed their OMH.

As it is the best practice to start administration of HAART almost immediately after the diagnosis of HIV infection, it is difficult if not impossible to differentiate between HAART induced HIV-OMH and idiopathic HIV-OMH.

To further complicate the statistics of surveying a particular population for HIV-OMH, it is well known that smokers have a high frequency of HIV-OMH than nonsmokers, and so smokers' melanosis sometimes may be mistaken for HIV-OMH.
